# A comparative study of lung toxicity in rats induced by three types of nanomaterials

**DOI:** 10.1186/1556-276X-8-521

**Published:** 2013-12-09

**Authors:** Zhiqing Lin, Li Ma, Zhu-ge X, Huashan Zhang, Bencheng Lin

**Affiliations:** 1 Institute of Health and Environmental Medicine, Academy of Military Medical Sciences, Tianjin 300050, People's Republic of China; 2 The Third People's Hospital of Datong, Datong, Shanxi Province 037008, People's Republic of China

**Keywords:** SWCNTs, Nano-Fe_3_O_4_, Nano-SiO_2_, BALF, Comparative proteomics analysis, Lung toxicity

## Abstract

The public is increasingly exposed to various engineered nanomaterials because of their mass production and wide application. Even when the biological effects of nanomaterials have been assessed, the underlying mechanisms of action *in vivo* are poorly understood. The present study was designed to seek a simple, effective, and oxidative stress-based biomarker system used for screening toxicity of nanomaterials. Nano-ferroso-ferric oxide (nano-Fe_3_O_4_), nano-silicon dioxide (nano-SiO_2_), and single-walled carbon nanotubes (SWCNTs) were dispersed in corn oil and characterized using transmission electron microscopy (TEM). Rats were exposed to the three nanomaterials by intratracheal instillation once every 2 days for 5 weeks. We investigated their lung oxidative and inflammatory damage by bronchoalveolar lavage fluid (BALF) detection and comparative proteomics by lung tissue. Two-dimensional electrophoresis (2-DE) of proteins isolated from the lung tissue, followed by matrix-assisted laser desorption-ionization time-of-flight mass spectrometry, was performed. In the present study, we chose to detect lactate dehydrogenase, total antioxidant capacity, superoxide dismutase, and malondialdehyde as the biomarker system for screening the oxidative stress of nanomaterials and IL-6 as the inflammatory biomarker in BALF. Proteomics analysis revealed 17 differentially expressed proteins compared with the control group: nine were upregulated and eight were downregulated. Our results indicated that exposure by intratracheal instillation to any of the three typical nanomaterials may cause lung damage through oxidative damage and/or an inflammatory reaction.

## Background

Nanomaterials are nanometer-sized materials with specific physicochemical properties that are different from those of micromaterials of the same composition. In recent years, as nanotechnology and materials science have progressed, engineered nanomaterials have been mass produced and widely applied. They are now routinely used as coating materials, cosmetic pesticides, and medications [1,2]. This means people are increasingly exposed to various kinds of manufactured nanoparticles in production and daily life. While nanomaterials provide benefits to diverse scientific fields, they also pose potential risks to the environment and to human health [3,4]. However, most studies have focused on the effects of one single type of particle or several particle types of the same substance, for example, nanoparticles and carbon nanotubes (CNTs) as carbonaceous nanomaterials. Rare studies have compared the toxicological effects of different types of nanomaterials, including carbonaceous, siliceous, and metal oxide nanoparticles. Because we are extremely lacking in epidemiological data on human exposure and health effects of nanomaterials at present, it is probably meaningful to elucidate this question for preventive sanitary control and health supervision during the creation and production of nanomaterials with special parameters. Particle size is a critical parameter which plays an essential role in the biological effects when concerning various types of nanoparticles with different shapes and composition. Therefore, a comparative study on the toxic effects of nanomaterials with varying properties seems to be necessary.

To date, animal studies have confirmed pulmonary inflammation, oxidative stress, and distal organ damage upon respiratory exposure to nanoparticles [5-8]. *In vitro* studies have also supported the physiological response found in whole-animal models and provide further data indicating the incidence of oxidative stress in cells exposed to nanoparticles. In recent years, the majority of toxicological response studies on nanomaterials have focused on cell culture systems [9,10]. However, data from these studies require verification from *in vivo* animal experiments. An understanding of toxicokinetics (the relationship between the physical properties of the nanomaterials and their behavior *in vivo*) would provide a basis for evaluating undesirable effects. Moreover, toxicoproteomics may identify predictive biomarkers of nanotoxicity. Although the biological effects of some nanomaterials have been assessed, the underlying mechanisms of action *in vivo* are little understood. We hypothesized that protein molecules were involved in the harmful effects of nanomaterials.

In this study, we used a consistent set of *in vivo* experimental protocols to study three typical nanomaterials that are characterized by particle size, shape, and chemical composition: single-walled carbon nanotubes (SWCNTs), silicon dioxide (SiO_2_), and magnetic iron oxide (Fe_3_O_4_) nanoparticles. We investigated their lung oxidative and inflammatory damage by bronchoalveolar lavage fluid (BALF) detection using biochemical analysis and comparative proteomics to the lung tissue. Two-dimensional electrophoresis (2-DE) of proteins isolated from the lung tissue, followed by matrix-assisted laser desorption-ionization time-of-flight (MALDI-TOF) mass spectrometry, was performed. The objectives were to explore the relationship between the comparable properties and the viability response of lung damage treated *in vivo* with different manufactured nanoparticles and to investigate the mechanism and markers of nanotoxicity in lung injury using biochemistry analysis in BALF and comparative proteomics in lung tissue.

## Methods

### Particle preparation

**Figure 1:**
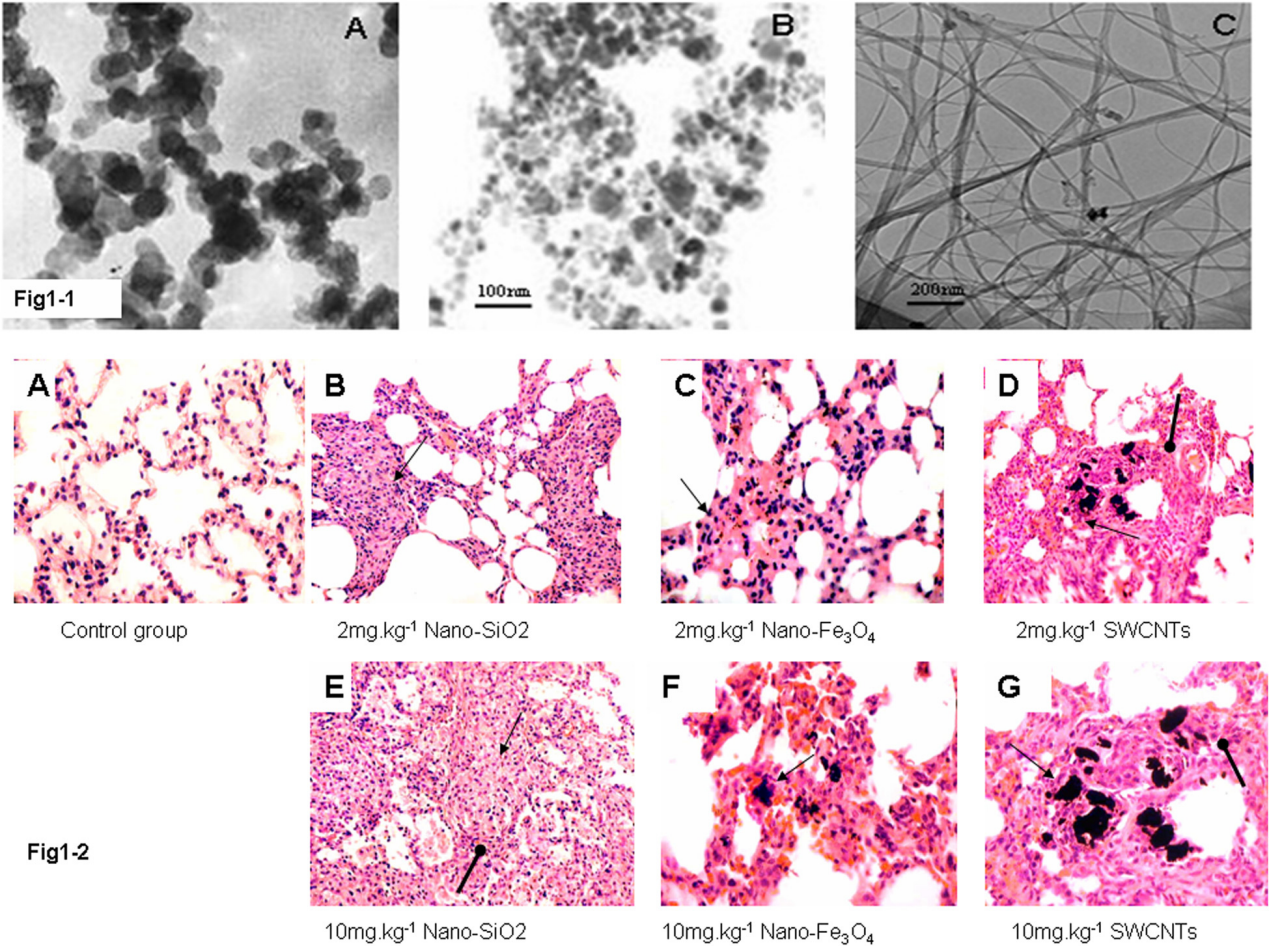
**Images of nanoparticles and lung tissue. ***1-1*: TEM images of engineered nanoparticles **(A)** SiO_2_, **(B)** Fe_3_O_4_, and **(C)** SWCNTs. *1-2*: Lung tissue from rats instilled with 2 (top) and 10 mg/kg (bottom) of a test material and euthanized 5 weeks after the single treatment. **(A)** Control group, **(B)** 2 mg/kg and **(E)** 10 mg/kg nano-SiO_2_, and **(C)** 2 mg/kg and **(F)** 10 mg/kg nano-Fe_3_O_4_. Particles were scattered in alveoli, and granulomas contained black particles (peaky arrow). **(D)** 2 mg/kg and **(G)** 10 mg/kg SWCNTs. An aggregate of inflammation cells (lymphocytes) (rotund arrow) around an area surrounded by quartz particle-containing, brown pellets were scattered in lung tissue. Magnifications were × 156.

Manufactured nanoparticles of SiO_2_, Fe_3_O_4_, and SWCNTs were purchased from commercial suppliers (Table 1). The particles were sterilized for 4 h at 180°C in an oven and then suspended in corn oil. To break the agglomerate and ensure a uniform suspension, all particle samples were sonicated six times intermittently (30 s every 2 min) and characterized using transmission electron microscopy (TEM) (JEM-100CX, JEOL Ltd., Tokyo, Japan). The size and shape of nanoparticles were summarized in Table 1. SiO exhibited a crystal structure with an average size of 30.2 nm (Figure 1 (1-1A)). Fe_3_O_4_ showed a sphere structure with an average size of 22.6 nm (Figure 1 (1-1B)). SWCNTs were rope-shaped with lengths less than 5 μm and diameters of approximate 8 nm (Figure 1 (1-1C)). The chemical composition was quantitatively analyzed by Raman spectroscopic technique, and the results show that the purities of the three nanomaterials are all more than 99.0%.

**Table 1:** Characterization on particle parameters of three typical nanomaterials

**Particles**	**Supplier**	**Size**	**Shape**	**Composition**
SWCNTs	COCC, Chinese Academy of Science, Chengdu, China	Diameter 8 nm; length <5 μm	Rope-shaped	C > 99.99%
Nano-SiO_2_	Runhe Co. Ltd, Shanghai, China	30.2 ± 9.4 nm	Crystal structure	SiO_2_ > 99.0%
Nano-Fe_3_O_4_	Nauno Co. Ltd, Shenzhen, China	22.6 ± 6.4 nm	Sphere	Fe_3_O_4_ > 99.0%

### Experimental animals and exposure to nanoparticles

Forty-nine SPF male (28) and female (21) Wistar rats weighing 180 to 210 g were used in compliance with the local ethics committee. Wistar rats (~200 g) were obtained from the Animal Center of the Academy of Military Medical Sciences (AMMS). Rats were housed in polycarbonate cages and kept on a 12-h light/dark cycle. Food and water were provided *ad libitum*. They were cared for and used humanely according to the Animal Care and Use Program Guidelines of AMMS. The rats were randomly divided into seven groups (each group had four male rats and three female rats in two cages, respectively, to avoid mating): the control group, SWCNTs low dose and high dose, nano-silicon dioxide (nano-SiO_2_) low dose and high dose, and nano-ferroso-ferric oxide (nano-Fe_3_O_4_) low dose and high dose. After anesthesia with ether, the rats were exposed to the nanomaterial suspension by intratracheal instillation with a dose of 2 and 10 mg/kg (body weight). The control group was treated with the same amount of corn oil. Every group received an intratracheal instillation once every 2 days for 35 days. After being anesthetized with 3% to 5% isoflurane in a small chamber, each rat was secured on an inclined plastic platform and anesthetization continued via a small nose cone. The trachea was exposed by a 1-cm hole for instillation of the nanomaterial suspension. Because it was difficult to eliminate air bubbles from the small nanomaterial-water sample drawn into the syringe, the intended volume, the test sample in a 200-μL aliquot, was drawn into a 1-mL syringe and rapidly propelled from the tubing and needle into the lungs. The rats were exposed to the nanomaterial suspension by intratracheal instillation once every 2 days for 5 weeks. The group of corn oil-instilled rats served as controls. After removal from the inhalation anesthetic, the rats recovered and were active within 10 min. The rats were divided into seven groups randomly by weight, including low-and high-dose groups of the three nanomaterials, and a control group.

### Histopathological evaluation

The middle of the left lungs was embedded in paraffin and thin-sectioned coronally; then, sections were stained with hematoxylin-eosin and examined by light microscopy.

### Preparation of BALF and detection

Twenty-four hours after the last instillation, rats were anesthetized with ether, bled from the femoral artery and sacrificed by cervical decapitation. The lung and trachea were exposed by dissection, and then the left lung was temporarily clamped. The right lung was lavaged with 6 mL of warm normal saline; then, the recovered BALF were centrifuged at 400 × *g* for 10 min. The concentrations of lactate dehydrogenase (LDH), total antioxidant capacity (T-AOC), superoxide dismutase (SOD), and malondialdehyde (MDA) in BALF were analyzed using biochemical analysis kits (Shangbo, Beijing, China). The reactions were measured using a UV/Vis spectrometer (UNICAM UV2, ATI-Unicam, Cambridge, UK). The levels of interleukin-1 (IL-1), interleukin-6 (IL-6), and tumor necrosis factor alpha (TNF-α) in lung homogenates were analyzed using enzyme-linked immunosorbent assay (ELISA) kits (Shangbo). The reactions were measured using an ELISA reader.

### Comparative proteomics analysis

The left lungs of rats were excised, immediately cooled in ice, and homogenized in a Teflon-glass homogenizer. Then, the homogenates were centrifuged at 700 × *g* for 15 min. Homogenized lung tissue of 40 to 100 mg was placed in 2 mL of lysis buffer containing 8 mol · L^−1^ urea, 4% CHAPS, 40 mmol · L^−1^ Tris, 65 mmol · L^−1^ DDT, and 1 mmol · L^−1^ PMSF and then centrifuged for 20 min at 12,000 rpm after being kept for 1 h at room temperature. Samples were stored in aliquots at −80°C. Protein determination was carried out according to the Bradford assay.

### Two-dimensional electrophoresis

#### First-dimension isoelectric focusing on immobilized pH gradient

One milligram of protein sample, 7 μL of DTT (1 mol · L^−1^), and 1.75 μL of IPG buffer (20 mmol · L^−1^) were solubilized in 350 μL of rehydration solution containing 8 mol · L^−1^ urea, 2% CHAPS, and a trace of bromophenol blue. This solution was pipetted into each 18-cm pH 3-10 strip holder. The strip holder was positioned on the IPGphor™ 3 isoelectric focusing system (Amersham Pharmacia, Little Chalfont, UK). Rehydration and isoelectric focusing (IEF) were carried out at 20°C. The current limit of each IPG strip was 50 μA. IEF was stopped at a maximum of 70,000 V · h^−1^.

#### Second-dimension SDS-PAGE of vertical slab

After IEF, the strips were equilibrated in a solution containing 50 mmol · L^−1^ Tris-HCL, 6 mol · L^−1^ urea, 30% glycerol, 2% SDS, and 1% DTT for 15 min, and then an additional 15 min in the same solution with 2.5% iodoacetamide substituted for 1% DTT.

After equilibration, vertical second-dimension separation was performed on 180 mm × 180 mm × 1 mm 13% homogeneous SDS-polyacrylamide gels. The IPG strips and low molecular weight protein markers were placed on gels and sealed using 0.5% agarose solution. Electrophoresis buffer containing 25 mmol · L^−1^ Tris base, 192 mmol · L^−1^ glycine, and 0.1% SDS was circulated at 16°C. The electrophoresis parameter of a strip was 20 mA × 40 min + 30 mA × 5 h. Electrophoresis was stopped when the bromophenol blue front was 1 mm from bottom of the gel. Coomassie brilliant blue R-250 staining was adopted for the gels.

### Image analysis

Image analysis was performed using ImageMaster software (Amersham Biosciences Little Chalfont, UK) according to the manufacturer's protocols. All values are expressed as the mean ± SD, and the difference in the abundance of protein spot was analyzed by a two-tailed *t* test. The level of significance was set at *p* < 0.05.

Stained gels were scanned using an image scanner, and images were processed using ImageMaster2D Elite (version 3.01) software. After spot detection and boundary average background subtraction, the gels were matched. For comparison, volumes of the protein spots were standardized. Student's *t* test was used to detect significant statistical differences in spot volume between the control and exposed groups (*p* < 0.05).

### In-gel digestion and protein identification by MALDI-TOF MS

The sodium dodecyl sulfate-polyacrylamide gel electrophoresis (SDS-PAGE) gel separated proteins were visualized by Coomassie Brilliant Blue G-250 staining. Selected differentially expressed protein spots were excised from preparative gels, and in-gel digestion was performed. The gel spots were washed three times with double distilled water and destained with a fresh solution containing 100 mM NH_4_HCO_3_ in 50% acetonitrile at 37°C. After being vacuum-centrifuged, the gel pieces were incubated in 10 μL of digestion solution consisting of 40 mM NH_4_HCO_3_ in 9% acetonitrile solution, and 20 μg/mL proteomic grade trypsin (Promega, Madison, WI, USA) for 10 to 12 h at 37°C.

In peptide mass fingerprint (PMF) map database searching, Mascot Distiller software was used to determine the monoisotopic peak list from the raw mass spectrometry files. Peptide matching and protein searches against the NCBI nonredundant (nr) databases were performed using the Mascot search engine (http://www.matrixscience.com) with a mass tolerance of ±0.3 Da. One missed coverage site was allowed for trypsin digestion, cysteine carbamidomethylation was assumed as a fixed modification, and iodoacetamide modification for cysteine and methionine oxidation was variable. Proteins were considered as identified only when they had a protein score ≥56, and results with C.I. % (confidence interval %) value >95% were considered to be a positive identification. The identified proteins were then matched to specific biological processes or functions by searching gene ontology using Uniprot/Swissprot database.

Protein spots were excised from 2-D gels, cut into 1 mm^3^, and destained by washing in a 100-μL solution containing 50% ACN and 25 mol · L^−1^ ammonium bicarbonate. The samples were then dried in a centrifugal evaporator for 20 min. Five microliters of trypsin solution (0.01 μg/μL containing 25 mol · L^−1^ ammonium bicarbonate) was added to the gel pieces and placed for 20 min at 4°C before incubating overnight at 37°C. Peptides were extracted by the addition of 40 μL of 2.5% TFA and 50% ACN. The two extraction volumes were incorporated and MALDI-TOF MS (Reflex III, Micromass, UK) was performed.

### Database searching

PMF from MALDI-TOF MS was used to search the NCBI nr protein database using the Mascot searching tool on MOWSE (11). Searching was performed using a missed cleavage site of one and a peptide mass tolerance of at most ±0.5 Da. Variable modifications were considered carbamidomethyl and/or oxidation (Table 2).

**Table 2:** Mascot result of significantly altered spots

**Spot**	**Protein name**	**Score**	**Change**	**Function**
1	Macrophage-capping protein	97	↑	Immunity
17	IgE-dependent histamine-releasing factor	81	↑	Immunity
12	Heat shock 27 kDa protein 1	104	↑	Immunity
2	Inward rectifier potassium channel protein IRK 3	72	↓	Ion channel
4	Potassium voltage-gated channel subfamily A member 3	91	↓	Ion channel
13	Glutathione peroxides 1	109	↓	Oxidation stress
10	Glutathione *S*-transferase alpha 5	63	↑	Oxidation stress
8	Glutathione transferase	88	↑	Oxidation stress
11	Ubiquinol-cytochrome-c reductase	79	↓	Metabolism
3	ATP synthase subunit alpha	83	↓	Metabolism
7	ADP/ATP transport protein	72	↓	Metabolism
9	Ca^2+^-transporting ATPase	94	↓	Metabolism
5	Phosophatidylethanolamine binding protein (TOF-TOF)	177	↑	Signal transduction
14	Annexin A11 (TOF-TOF)	89	↑	Signal transduction
15	GTP-binding protein Rab40c	90	↑	Signal transduction
16	Protein-tyrosine-phosphatase isoenzyme AcP1	117	↑	Signal transduction
6	Transgelin 2 (Sm22 alpha) (TOF-TOF)	121	↑	Cytoskleton

The table shows putative protein identifications of significantly altered protein spots isolated from lung samples of rats exposed to three types of nanomaterials based on peptide mass fingerprint (PMF) map database searching using Mascot Distiller software.

### RT-PCR

Total RNA was isolated by the acid guanidium thiocyanate-phenol-chloroform method using the Isogen reagent (Nippon Gene, Tokyo, Japan) from pulverized frozen left lung parenchyma (Fisher Scientific, Suwanee, GA, USA) in liquid nitrogen and then treated with RNase-free DNase. RNA concentration was determined by ultraviolet (UV) light absorbance at 260 nm. For RT-PCR analysis, first-strand cDNA synthesis was completed from total RNA (5 μg) with the SuperScript™ First-Strand Synthesis System for RT-PCR (Invitrogen Co., Carlsbad, CA, USA) and Oligo(dT) primer. Primer sequences, generated using GenBank searches with BLASTN, were used to generate PCR products using Taq DNA polymerase (TaKaRa Ex Taq™ Takara Bio Inc., Kyoto, Japan) and an iCycler thermocycler (Bio-Rad Laboratories, Inc., Hercules, CA, USA). Pilot studies were performed to determine the optimal annealing temperature and to confirm a linear correlation between the number of PCR cycles and the densitometric intensity of amplicons. Samples were analyzed for genomic DNA contamination by PCR analysis of total RNA. PCR products were size-separated by electrophoresis on 2% agarose gel, visualized by ethidium bromide staining under UV light, and analyzed by scanning densitometry. Results were expressed as density of transgelin 2 in relation to β-actin, an internal control, expression within the same sample.

### Western blotting

Western blot detection of transgelin 2 and the internal control β-actin, was performed using standard protocols. In detail, lung tissue specimens from all subjects were homogenized to obtain protein extracts. The protein lysate was added to one-third volume of the SDS preparation buffer (NuPAGE 4× LDS Sample Buffer, Invitrogen Corp.). These protein samples (50 μg) were separated by 12.5% SDS-polyacrylamide gel electrophoresis. The proteins were then transferred electrophoretically to nitrocellulose membranes, which were incubated with a mouse anti-transgelin 2 monoclonal antibody (Santa Cruz Biotechnology Inc., Santa Cruz, CA, USA). After secondary antibody application, immunodetection was performed by enhanced chemiluminescence on X-ray films (Fuji films). The mouse anti-actin antibody (MAB 1501, Chemicon, Temecula, CA, USA) was used to normalize transgelin 2 expression. Films were scanned and the protein lanes were quantified using Photoshop CS2 image analysis software (Adobe Systems Inc., San Jose, CA, USA).

## Results

### Characteristics of the three nanomaterials

The size and shape of nanoparticles were summarized in Figure 1 (1-1). Our characterizations indicated that SiO_2_ nanoparticles exhibited a crystal structure with an average size of 20.2 nm (Figure 1 (1-1A)), that Fe_3_O_4_ nanoparticles had a sphere shape with an average size of 40 nm (Figure 1 (1-1B)), and that CNTs were rope-shaped with lengths <5 μm and diameters of approximately 8 nm (Figure 1 (1-1C)). Each chemical composition was quantitatively analyzed using a Raman spectroscopic technique and showed a purity >99.0% for all three nanomaterials.

### Pathological observations of the lung

Histopathological evaluation of lung tissues revealed that pulmonary exposures to nanoparticles in rats produced persistent and progressive lung inflammatory responses. As shown in Figure 1 (1-2), the SWCNTs caused an overall change in alveolar architecture with the exception of focal collections of alveolar macrophages laden with particles; lung tissue thickening as a prelude to the development of fibrosis was evident and progressive (Figure 1 (1-2D,G)), and such effects were absent in the lungs of the control group. The lungs of the SiO_2_ and Fe_3_O_4_ groups also produced mild to moderate alveolar and interstitial inflammation; inflammation cells were predominately inside the edema area, and none were in the area of normal alveolar tissue in the lungs of the control group (Figure 1 (1-2B,C,E,F)).

### Concentrations of LDH, T-AOC, SOD, and MDA in BALF

After 35 days of intratracheal instillation, LDH, T-AOC, SOD, and MDA values were measured in BALF as indicators of oxidative damage in the lungs of nanomaterial-exposed rats. Compared with the control group, the levels of LDH and MDA were both increased (*p* < 0.05) with T-AOC and SOD decreasing (*p* < 0.05) with a high dose of the three nanomaterials in the exposed groups. There were some differences among the three nanomaterials: At both doses of 2 and 10 mg/kg of nanomaterials, the activity of T-AOC and SOD in SWCNT-exposed rats was lower than that in nano-SiO_2_- and nano-Fe_3_O_4_-exposed rats (*p* < 0.05); however, at a high dose of 10 mg/kg of nanomaterials, the activity of LDH and MDA in SWCNT-exposed rats was higher than that in nano-SiO_2_- and nano-Fe_3_O_4_-exposed rats (*p* < 0.05) (Table 3). Moreover, Table 3 also showed that the activity of T-AOC and SOD in nano-SiO_2_-exposed rats was lower than that in nano-Fe_3_O_4_-exposed rats (*p* < 0.05).

**Table 3:** Concentrations of LDH, T-AOC, SOD, and MDA in BALF

**Groups**	**LDH (U.g.prot**^**−1**^**)**	**T-AOC (U.mg.prot**^**−1**^**)**	**SOD (U.mg.prot**^**−1**^**)**	**MDA (nmol.mL**^**−1**^**)**
Control group	609.24 ± 109.88	8.95 ± 0.48	8.95 ± 0.48	0.87 ± 0.32
2 mg.kg^−1^ nano-Fe_3_O_4_	651.58 ± 162.60	7.62 ± 0.39^a^	7.62 ± 0.39^a^	1.15 ± 0.39
2 mg.kg^−1^ nano-SiO_2_	752.62 ± 181.74	7.04 ± 0.86^a^	7.03 ± 0.86^a^	1.22 ± 0.27
2 mg.kg^−1^ SWCNTs	796.84 ± 157.01	4.87 ± 0.47^a,b,c^	5.01 ± 0.37^a,b,c^	1.35 ± 0.69
10 mg.kg^−1^ nano-Fe3O_4_	770.00 ± 109.78^a^	7.74 ± 0.76^a,c^	7.03 ± 0.43^a,c^	2.05 ± 0.44^a^
10 mg.kg^−1^ nano-SiO_2_	786.65 ± 116.70^a^	5.61 ± 0.95^a,b^	6.18 ± 0.46^a,b^	2.43 ± 0.79^a^
10 mg.kg^−1^ SWCNTs	1,084.18 ± 200.36^a,b,c^	4.13 ± 0.29^a,b,c^	4.28 ± 0.41^a,b,c^	4.15 ± 0.52^a,b,c^

^a^Compared with the control group, *p* < 0.05. ^b^Compared with the nano-Fe_3_O_4_ group at the same dose, *p* < 0.05. ^c^Compared with the nano-SiO_2_ group at the same dose, *p* < 0.05.

### Concentrations of IL-6, IL-1, and TNF-α in BALF

After 35 days of intratracheal instillation, the levels of IL-6 in BALF among the rats exposed to the three nanomaterials were greater than those of the control group (*p* < 0.05), as well as the level of TNF-α in a high dose of 10 mg/kg nano-SiO_2_ and SWCNTs. In addition, in a dose of 10 mg/kg, the level of TNF-α of nano-SiO_2_- and SWCNTs-exposed rats was greater than that of nano-Fe_3_O_4_-exposed rats (Table 4).

**Table 4:** Concentrations of IL-1, IL-6, and TNF-α in BALF

**Groups**	**IL-1 (pg.mL**^**−1**^**)**	**IL-6 (pg.mL**^**−1**^**)**	**TNF-α (pg.mL**^**−1**^**)**
Control group	12.68 ± 3.73	23.55 ± 4.57	12.61 ± 1.96
2 mg.kg^−1^ nano-Fe_3_O_4_	10.63 ± 3.72	34.75 ± 2.28^a^	13.23 ± 1.35
2 mg.kg^−1^ nano-SiO_2_	12.32 ± 4.77	29.80 ± 5.00^a^	13.62 ± 1.82
2 mg.kg^−1^ SWCNTs	9.34 ± 2.40	34.21 ± 6.73^a^	13.66 ± 1.72
10 mg.kg^−1^ nano-Fe_3_O_4_	10.05 ± 1.76	40.59 ± 10.56^a^	13.36 ± 1.41
10 mg.kg^−1^ nano-SiO_2_	14.76 ± 4.16	33.21 ± 5.80^a^	17.72 ± 1.80^a,b^
10 mg.kg^−1^ SWCNTs	10.11 ± 3.07	42.92 ± 16.20^a^	17.08 ± 1.35^a,b^

^a^Compared with control group, *p* < 0.05. ^b^Compared with the nano-Fe_3_O_4_ group at the same dose, *p* < 0.05.

### Comparative proteomic comparisons

**Figure 2:**
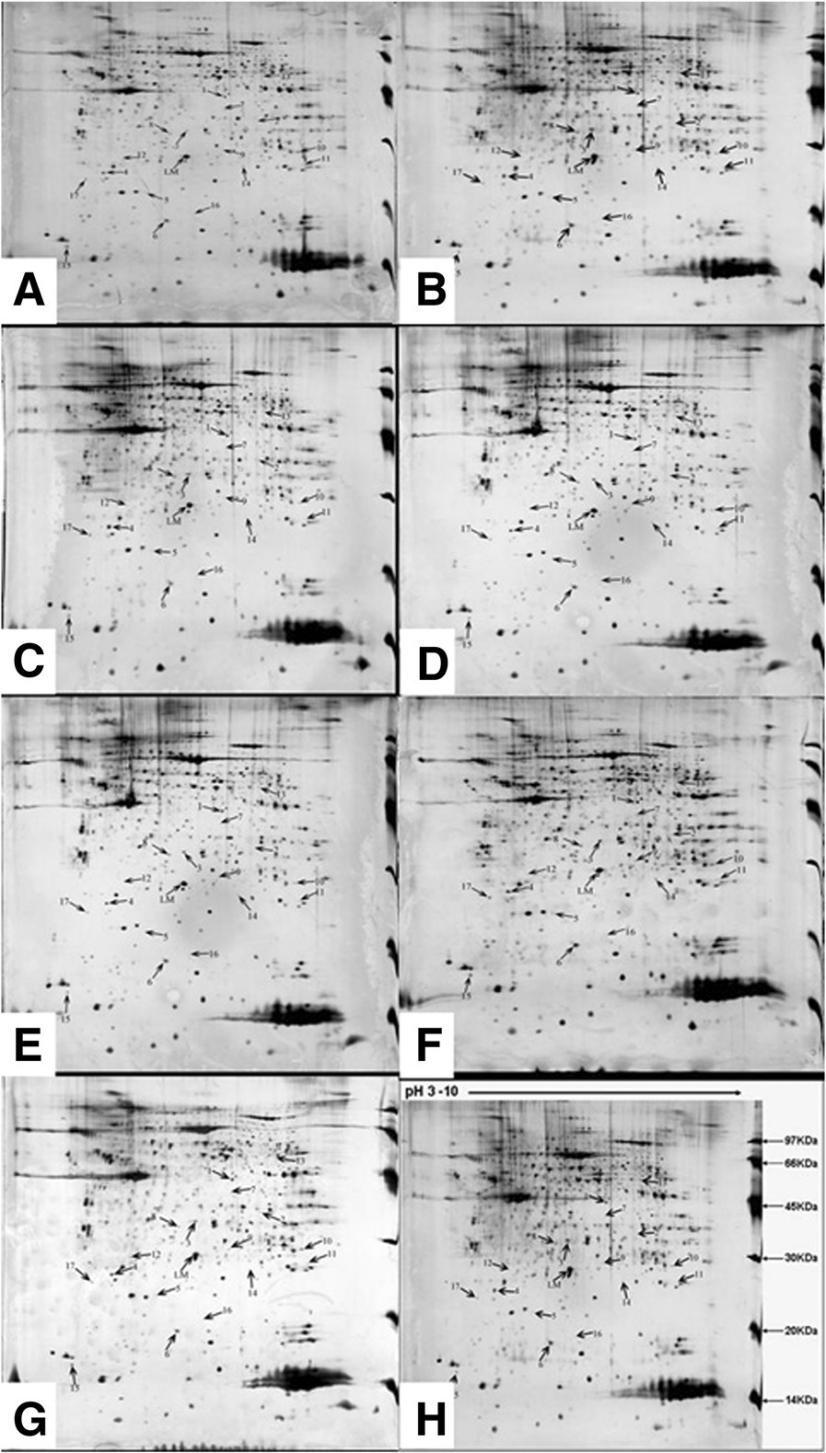
**2-DE maps of lung proteins.** From rats of the **(A)** control and **(B-G)** nanomaterial-treated groups (**B**, 2 mg/kg nano-Fe_3_O_4_; **C**, 2 mg/kg nano-SiO_2_; **D**, 2 mg/kg SWCNTs; **E**, 10 mg/kg nano-Fe_3_O_4_; **F**, 10 mg/kg nano-SiO_2_; **G**, 10 mg/kg SWCNTs). **(H)** Protein solutions from the lungs of male and female rats were separated by IEF (linear pH gradient from pH 3 to 10) and SDS-PAGE (13% polyacrylamide gel) methods, respectively . Each gel is representative of three independent replicates. There are 17 differentially expressed proteins from the rat lung, each marked by an arrow and number. The match rate of protein is over 72.2% among the samples at the same group.

The separation of 1.5 mg of protein samples resulted in 2-D spot patterns. Approximately 850 protein spots could be visualized at a pH of 3 to 10 and Mr 14,400 to 97,000, and their normalized volumes were compared statistically. Of these proteins, 17 were significantly altered compared with the control group (*p* < 0.05), but there was no difference between different nanomaterial-exposed groups at the different doses (Figure 2). The relative volumes of these spots are listed in Table 5.

**Table 5:** Relative volumes of significantly altered protein spots isolated from lung samples of rats

**Spot**	**Control**	**H-nano-SiO**_**2**_	**L-nano-SiO**_**2**_	**H-nano-Fe**_**3**_**O**_**4**_	**L-nano-Fe**_**3**_**O**_**4**_	**H-SWCNTs**	**L-SWCNTs**
1	0.103 ± 0.020	0.195 ± 0.019^a^	0.184 ± 0.012^a^	0.162 ± 0.016^a^	0.172 ± 0.014^a^	0.160 ± 0.026^a^	0.194 ± 0.033^a^
2	0.087 ± 0.020	0.024 ± 0.011^a^	0.012 ± 0.003^a^	0.027 ± 0.008^a^	0.039 ± 0.014^a^	0.020 ± 0.010^a^	0.026 ± 0.005^a^
3	0.330 ± 0.039	0.128 ± 0.021^a^	0.182 ± 0.030^a^	0.200 ± 0.038^a^	0.143 ± 0.016^a^	0.140 ± 0.015^a^	0.182 ± 0.059^a^
4	0.356 ± 0.049	0.203 ± 0.015^a^	0.215 ± 0.022^a^	0.226 ± 0.011^a^	0.231 ± 0.026^a^	0.201 ± 0.023^a^	0.208 ± 0.019^a^
5	0.014 ± 0.006	0.032 ± 0.008^a^	0.030 ± 0.006^a^	0.031 ± 0.005^a^	0.032 ± 0.004^a^	0.040 ± 0.005^a^	0.031 ± 0.003^a^
6	0.193 ± 0.030	0.405 ± 0.047^a^	0.382 ± 0.045^a^	0.404 ± 0.044^a^	0.400 ± 0.050^a^	0.434 ± 0.024^a^	0.400 ± 0.037^a^
7	0.036 ± 0.007	0.012 ± 0.001^a^	0.017 ± 0.003^a^	0.012 ± 0.002^a^	0.017 ± 0.001^a^	0.012 ± 0.002^a^	0.013 ± 0.003^a^
8	0.053 ± 0.020	0.151 ± 0.020^a^	0.136 ± 0.044^a^	0.146 ± 0.021^a^	0.137 ± 0.007^a^	0.140 ± 0.029^a^	0.140 ± 0.013^a^
9	0.038 ± 0.006	0.016 ± 0.004^a^	0.017 ± 0.003^a^	0.017 ± 0.003^a^	0.019 ± 0.007^a^	0.010 ± 0.008^a^	0.013 ± 0.007^a^
10	0.092 ± 0.028	0.257 ± 0.027^a^	0.245 ± 0.020^a^	0.228 ± 0.039^a^	0.219 ± 0.031^a^	0.264 ± 0.040^a^	0.214 ± 0.029^a^
11	0.098 ± 0.013	0.016 ± 0.004^a^	0.024 ± 0.006^a^	0.018 ± 0.003^a^	0.023 ± 0.003^a^	0.013 ± 0.004^a^	0.022 ± 0.004^a^
12	0.030 ± 0.003	0.189 ± 0.051^a^	0.158 ± 0.036^a^	0.186 ± 0.044^a^	0.132 ± 0.022^a^	0.196 ± 0.027^a^	0.160 ± 0.044^a^
13	0.153 ± 0.020	0.031 ± 0.018^a^	0.059 ± 0.020^a^	0.045 ± 0.021^a^	0.070 ± 0.029^a^	0.040 ± 0.029^a^	0.054 ± 0.029^a^
14	0.012 ± 0.003	0.038 ± 0.008^a^	0.031 ± 0.007^a^	0.049 ± 0.009^a^	0.032 ± 0.005^a^	0.043 ± 0.009^a^	0.037 ± 0.007^a^
15	0.051 ± 0.008	0.135 ± 0.027^a^	0.109 ± 0.018^a^	0.126 ± 0.013^a^	0.122 ± 0.024^a^	0.147 ± 0.022^a^	0.114 ± 0.017^a^
16	0.021 ± 0.003	0.055 ± 0.007^a^	0.051 ± 0.012^a^	0.053 ± 0.011^a^	0.490 ± 0.007^a^	0.046 ± 0.008^a^	0.042 ± 0.004^a^
17	0.036 ± 0.009	0.088 ± 0.015^a^	0.079 ± 0.013^a^	0.105 ± 0.009^a^	0.0105 ± 0.025^a^	0.102 ± 0.030^a^	0.108 ± 0.015^a^

The rats were exposed to three types of nanomaterials: SiO_2_, Fe_3_O_4_, and SWCNTs. All the 17 spots have higher expression in the groups exposed to the three nanomaterials than in the control group (*p* < 0.05), and there is no significant difference between two doses of the same nanomaterial (*p* > 0.05). ^a^Compared with the control group, *p* < 0.05.

### MALDI-TOF MS and Mascot searching

**Figure 3:**
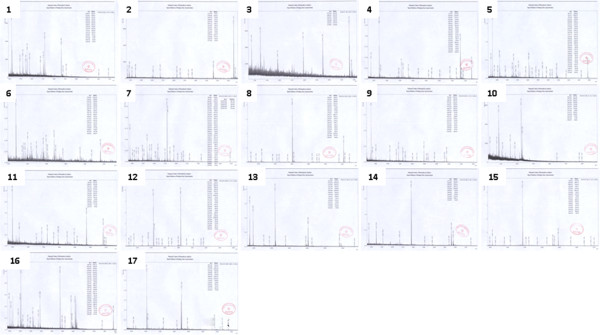
**The results of MALDI-TOF MS in 17 different spots.** The peptide mass fingerprinting of identified proteins were matched to specific biological processes or functions by searching Gene Ontology using Uniprot/Swissprot database.

Differentially expressed protein spots were *in situ* digested with trypsin and analyzed by MALDI-TOF and MALDI-TOF/MS. Using the Mascot search engine, 17 protein spots were successfully identified, 11 proteins in female rats, 5 proteins in male rats, and 1 protein (transgelin 2) both in female and male rats. The matched proteins in the database were mainly from *Rattus*. Analysis of the protein expression using ImageMaster 2D Platinum software and comparison of protein expression between nanomaterial-treated groups and control group were done. High-quality PMF, the MALDI-TOF/TOF mass spectrometry map, and database results are shown in Figure 3. The identified proteins were then matched to specific biological processes or functions by searching Gene Ontology (GO terms) using Uniprot/Swissprot database and submitted to Ingenuity Pathways Analysis. We classified these proteins manually to a variety of cellular biological processes, such as immunity, ion channel regulation, oxidative stress, metabolism, signal transduction, and cytoskeletal development.

### Quantitative real-time PCR analysis

**Figure 4:**
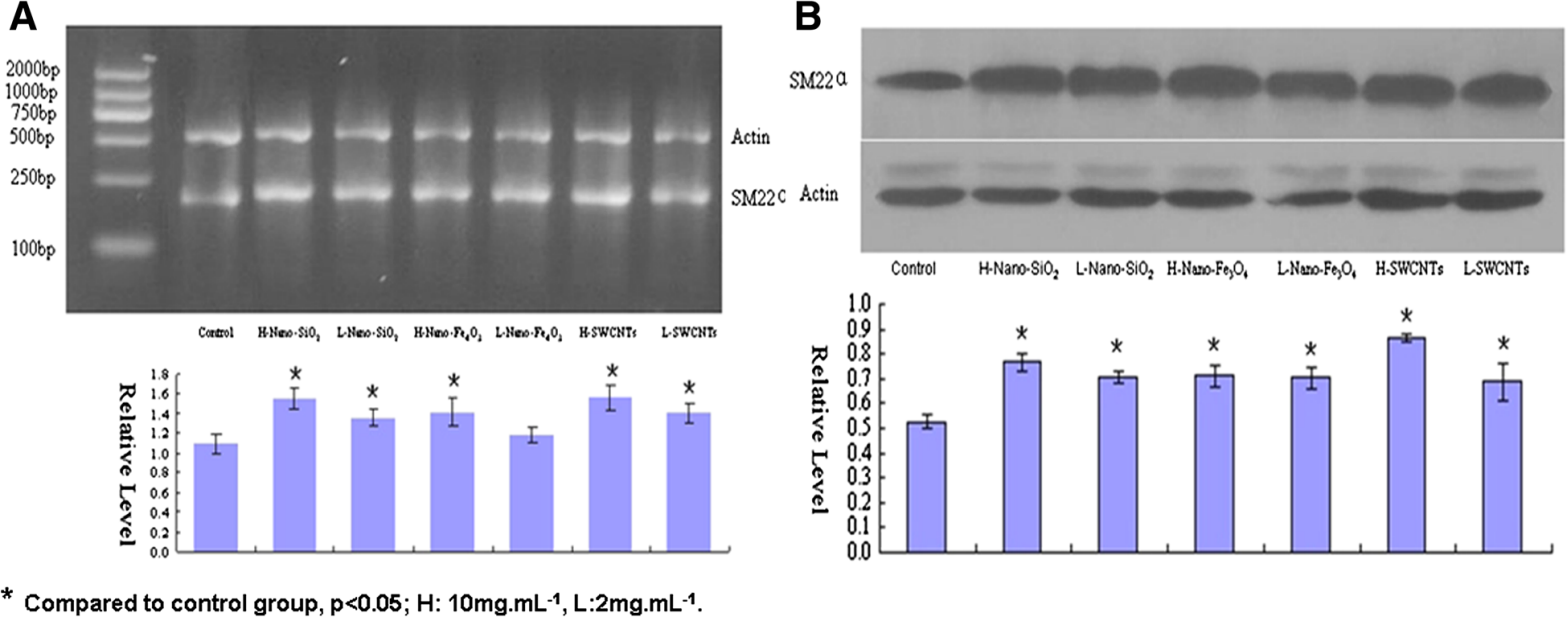
**Real-time PCR (A) and Western blot (B) analysis of selected genes: SM22α, Transgelin 2.** Bars represent the relative fold changes compared with controls. Error bars represent the S.E.M. for the average fold changes. Statistical significance (*p* < 0.05) between expression following nanomaterial exposure and the controls is denoted by an asterisk (*).

Transgelin 2 gene expression was also named SM22α, analyzed by quantitative PCR across all treatment groups (nano-SiO_2_, nano-Fe_3_O_4_, SWCNTs) (Figure 4A). Transgelin 2 gene expression was significantly increased at all doses of nanomaterial exposure, except low-dose nano-Fe_3_O_4_, compared with the control group (*p* < 0.05), but the majority of nanomaterial groups showed almost no significant difference between high-dose and low-dose groups. Transgelin 2 mRNA levels were increased the most by high-dose SWCNT exposure.

### Western blot analysis

Transgelin 2 protein was analyzed by Western blot in all treatment groups (nano-SiO_2_, nano-Fe_3_O_4_, SWCNTs) (Figure 4B). Transgelin 2 protein expression was significantly increased at all doses of nanomaterial exposure compared with the control group (*p* < 0.05), but there was almost no significant difference between high dose and low dose in nanomaterial exposure groups.

## Discussion

A nanomaterial is a kind of ultrafine material composed of nanosized particles, between 0.01 and 100 nm in diameter. Recently, research and development of these particles have increased [11], and their potential adverse effects are being investigated by researchers around the world [12-14]. Some report that ultrafine particles may cause damage to the body due to their higher activity and selectivity [13]. The effects of ultrafine particles on the lungs have received much more attention. In spite of the lungs being the most direct target organ for such particles, the methods to study lung injury are limited except for histopathobiology, so we attempt to use biochemical analysis and comparative proteome to detect lung damage *in vivo* after nanomaterial exposure to find the difference between the nanomaterials and non-nanomaterials.

We selected the three typical nanomaterials because of their different chemical compositions (nano-SiO_2_ is an inorganic oxide, nano-Fe_3_O_4_ is a metal oxide, and SWCNT is a carbon) and different shapes (nano-SiO_2_ has a crystal structure, nano-Fe_3_O_4_ is a sphere, and SWCNT is rope-shaped). In our study, we found that the three nanomaterials induced oxidative damage and inflammation in BALF. In addition, there are 17 different proteins regardless of the composition and shape of nanomaterials which expressed a similar nanosize.

Epidemiologic and experimental animal studies have shown an increased risk of respiratory and cardiovascular morbidity and mortality associated with exposure to ultrafine particles [15,16]. Nanoparticle exposure induced production of cytokines in lung epithelial cells and in lung tissue [17,18]. The aim of this study was to characterize the biochemical changes in BALF and protein profiles in the lung tissue of rats following exposure to three nanomaterials using newly available technologies especially comparative proteomics.

Higher protein concentrations in the nanomaterial-exposed BALF samples are likely a result of plasma extravasation. Consistent with this view, many of the plasma-derived proteins identified in both exposed and control samples do indeed change in abundance, for example, albumin [17], but additional work will be required to provide accurate quantification. Proteinase digestion may have reduced protein levels in lung samples from exposed animals, or the presence of proteins may have been masked by the higher total protein concentration in these samples.

In this study, SWCNT induced the strongest oxidative damage in BALF among the three nanomaterials (Tables 3 and 4). LDH leakage is a measure of toxicity on the basis of membrane integrity damage. All three types of nanomaterials induced apparent LDH leakage in BALF, which revealed the impact of nanoparticles on cell membrane integrity. Compared with the controls, LDH levels in BALF were gradually elevated as particle concentrations increased. Following exposure to SWCNTs, SiO_2_, and Fe_3_O_4_ at the highest dosage levels, LDH releases were increased by 77.9%, 29.1%, and 26.4%, respectively, significantly higher than the untreated control (*p* < 0.05). The effect was also significant as that on MDA. In addition, it was noted that no statistically significant difference was found when comparing the effects among different types of nanoparticles at the low-dosage level. Furthermore, the decreases of T-AOC and SOD values in exposed groups suggested that the balance between oxidation and anti-oxidation was destroyed in rats. In addition, SWCNTs exhibited greater lung damage than SiO_2_ and Fe_3_O_4_ nanoparticles at a high dosage which elicited more oxidative stress. It probably suggested that the acute toxicity primarily originated from the cellular internalization of nanoparticles rather than physical damage on the cellular membrane. ELISA was employed to determine the protein concentrations of TNF-α, IL-6, and IL-1 in BALF of rats. Cytokines play an important role in regulating immunity and are classified into proinflammatory (TNF-α, IL-6, and IL-1) and anti-inflammatory (IL-10, IL-4, and IL-13). As proinflammatory factors, the level of IL-6 induced by the nanomaterials in BALF was significantly higher than that of the control group, but the level of IL-1 induced by nanomaterials was not significantly different compared to control group. However, the level of TNF-α induced by nano-SiO_2_ and SWCNTs at a high dosage showed significant difference compared to the control group and nano-Fe_3_O_4_-exposed rats. This was in accordance with the results obtained from the histopathological evaluation of lung tissues which revealed that pulmonary exposures to nanoparticles in rats produced persistent and progressive lung inflammatory responses. The presence of an inflammatory response is further supported by the qualitative analysis of the proteins identified by liquid chromatography/mass spectrometry (LC/MS). Nanomaterial-exposed samples in our study showed a pronounced increase in the amount and number of proteins observed, which appears to be caused by damage at the air-blood barrier [19-22].

The spectra obtained using a MALDI-TOF-MS Reflex III contained 17 readily observable peaks that were specific to lung samples taken from rats after exposure to nanomaterials. Subsequent analysis using LC/MS indicates that the proteins producing these peaks are related to immunity response proteins (macrophage-capping protein, IgE-dependent histamine-releasing factor, and heat shock 27 kDa protein 1 were upregulated), ion channel proteins (inward rectifier potassium channel protein IRK3 and potassium voltage-gated channel subfamily A member 3 were downregulated), oxidative stress proteins (glutathione peroxidase 1 was upregulated; glutathione *S*-transferase alpha-5, glutathione transferase, and ubiquinol-cytochrome-c reductase were downregulated), metabolism proteins (ATP synthase subunit alpha, ADP/ATP transport protein, and Ca^2+^-transporting ATPase were downregulated), signal transduction proteins (phosphatidylethanolamine-binding protein, annexin Al 1, GTP-binding protein Rab40c, and protein-tyrosine-phosphatase isoenzyme AcP1 were upregulated) and cytoskeleton proteins (transgelin 2 was upregulated). Seventeen proteins, most of which are lung damage and inflammation specific, repeatedly showed differential regulation in the nanomaterial-exposed samples compared with the control group.

Based on the proteins identified, the major observed effect of nanomaterial exposure is an inflammatory response. Macrophage-capping protein, IgE-dependent histamine-releasing factor, and heat shock 27 kDa protein 1, well-known mediators of inflammation, were upregulated. This is in accordance with the results obtained from the inflammatory factor in BALF and lung pathological analysis.

Glutathione transferase, glutathione *S*-transferase alpha-5, ubiquinol-cytochrome-c reductase, and glutathione peroxidase 1, all related to oxidative stress, were upregulated in the groups exposed to the three nanomaterials, indicating that the nanoparticles could induce the oxidative damage in lung tissue, which consumed considerable glutathione peroxidase to make the three enzymes of glutathione transferase, glutathione *S*-transferase alpha-5, and ubiquinol-cytochrome-c reductase accumulation to destroy the balance of oxidative and anti-oxidation.

ATP synthase subunit alpha, ADP/ATP transport protein, inward rectifier potassium channel protein IRK3, and Ca^2+^-transporting ATPase, all associated with ATP synthesis, were downregulated in the groups exposed to the three nanomaterials, indicating that the histiocytes of the lung were short of energy. Intratracheal instillation of nanomaterials injured lungs and influenced food intake, even nutrient absorption and metabolism, which was reflected in the decreased weight of nanomaterial-exposed rats.

These 17 different proteins were in concert with the results obtained from the biochemical assays in BALF which showed obvious diversity in oxidative and inflammatory damage of the three nanomaterials.

The discovery of transgelin 2 in the MALDI-TOF data provoked our interest, which also demonstrated an advantage to a top-down proteomics approach. Transgelin 2 is a marker of cell differentiation. Lung fibroblasts (LFs) only exist in normal lung tissue. After lung damage, LFs differentiate into myofibroblasts (MFs), which is identified by transgelin 2 in the cytoplasm. MS analysis of the cytoskeleton protein is unable to distinguish processed and unprocessed transgelin 2, which was positively expressed in the groups exposed to the three nanomaterials but negatively expressed in the control group. The SDS-PAGE analysis confirmed the MALDI-TOF data but was more difficult to perform. Proteins, such as the transgelin 2, may be a marker of carcinoma in the stomach and hepatomas. Thus, they play major biological roles and are important to be characterized. Currently, a combination of RT-PCR and Western blot analyses is required to verify proteome coverage. The result of RT-PCR indicated that the mRNA level of transgelin2 in the lung was respectively increased compared to the control group. The expression of transgelin 2 in the lung was indeed increased in the nanomaterial groups tested by Western blot, and this result further confirmed the result of 2-DE. The results indicate that transgelin 2 protein may be a biomarker of lung damage induced by nanomaterials.

Among these results, we found that SWCNTs had a greater toxicity compared to the other two nanomaterials. Besides chemical composition, other particle properties such as size and shape may also affect the addressed specific physicochemical and transport properties, with the possibility of negating amplification of the surface effects. Therefore, it is educible that the greatest damage caused by SWCNTs may come from mechanical injury and oxidative effect. It is likely that SWCNTs might penetrate the lung epidermic cell into the cell nucleus through nucleopores and then destruct the cell structure. To combine the above two points, the toxicity of different nanoparticles may primarily be due to particle shape rather than chemical composition. However, since the available techniques are really scarce at present, it is rather difficult to inspect intracellular translocation of nanoparticles. Unfortunately, we cannot directly confirm the actual process from our data.

We focused on SWCNTs, SiO_2_, and Fe_3_O_4_ nanoparticles as examples of typical manufactured nanomaterials that are associated with environmental and occupational exposure. These nanomaterials are produced on an industrial scale, serving as raw materials of printer toners, semiconductors, catalysts, and cosmetics. Previous studies have demonstrated that exposure to some types of nanoparticles induces toxicological effects in different cell lines and key organs in general. However, on account of lacking standard strategies and methods for toxicological evaluation on nanomaterials, it is rather difficult for us to decide which kind of nanomaterials may be a greater health hazard. Additionally, comparative studies which could provide useful references on this question are very sparse. In this study, we examined the effect of the three typical nanomaterials on rats' lungs. It is reasonable to suggest that according to our results, more attention should be paid to the biosafety evaluation of SWCNTs. Transgelin 2 should also be given sufficient attention. Finally, we have to point out that this investigation did not elucidate the particle state during reaction with organs, e.g., agglomeration, distribution, and metabolism because of the difficulties in present techniques.

## Conclusion

In summary, we demonstrate that it is possible to detect LDH, T-AOC, SOD, and MDA as biomarkers of oxidative damage and IL-6 as an inflammatory biomarker after nanoparticle exposure causes lung damage in rats using biochemical detecting systems. Comparative proteomics could be used as a high-throughput method to find the concordance, and mass spectrometry was used to identify the predominant peaks present in the MALDI-TOF spectra to provided additional proteins displaying differential responses to nanomaterial exposure. The results would provide the laboratory data for further studies in humans exposed to nanomaterials and nanosafety research.

## Competing interests

The authors declare that they have no competing interests.

## Authors’ contributions

ZQL and LM participated in the design of the study, carried out the experiments, and drafted the manuscript. BCL checked the manuscript grammar and modified the draft of the manuscript. HSZ performed the statistical analysis. ZGX designed the study and guided this work. All authors read and approved the final manuscript.
